# Adverse Effect of Lymph Node Dissection in Metastatic Renal Cell Cancer Patients Treated with Cytoreductive Nephrectomy: A Contemporary Analysis of Survival

**DOI:** 10.7150/jca.33923

**Published:** 2019-08-08

**Authors:** Dalong Cao, Yongqiang Huang, Chuankai Zhang, Junlong Wu, Leijun Yu, Hailiang Zhang, Guohai Shi, Dingwei Ye

**Affiliations:** 1 Department of Urology, Fudan University Shanghai Cancer Center, Shanghai 200032, China.; 2 Department of Oncology, Shanghai Medical College, Fudan University, Shanghai 200032, China.; 3 Department of Burn and Plastic Surgery, Ruijin Hospital, School of Medicine, Shanghai Jiao Tong University, Shanghai 200025, China.

**Keywords:** Metastatic renal cell cancer, Cytoreductive nephrectomy, Surveillance, Epidemiology and End Result program, Lymph node dissection, Cancer-specific survival, Overall survival

## Abstract

**Background and objectives:** In patients with metastatic renal cell cancer (mRCC), cytoreductive nephrectomy (CN) may occasionally be performed. However, the role of lymph node dissection (LND) for such cases is unknown in era of target therapy. To test the effect of LND at CN on cancer-specific survival (CSS), overall survival (OS) in era of target therapy compared with no LND in patients with mRCC.

**Methods:** A total of 4690 mRCC patients treated with CN were identified within the Surveillance, Epidemiology, and End Result (SEER) database (2006-2015). Survival differences were assessed by Kaplan-Meier estimate and compared using log-rank test. Multivariable Cox regression analysis (MCR) was used to evaluate the effect of LND on CSS and OS.

**Results:** Within the SEER database, 1902 (40.6%) of 4690 mRCC patients underwent LND at CN. MCR analysis showed that LND at CN exhibited lower CSS (hazard ratio [HR] 1.18, 95% confidence interval (CI) 1.09-1.27; p < 0.01) and OS (HR 1.13, 95% CI 1.05-1.21; p < 0.01) compared with non-LND in mRCC patients. The adverse effect of LND on CSS and OS were also detected in metastatic patients with clear cell RCC (ccRCC) and non-ccRCC (all p<0.0001). Additionally, the association of number of resected node with CSS (HR 0.98, 95% CI 0.88-1.10; p = 0.68) and OS (HR 1.00, 95% CI 0.89-1.11; p = 0.93) were not observed in MCR analysis.

**Conclusion:** We are the first to demonstrate that LND at CN is associated with poor CSS and OS in metastatic patients with ccRCC and non-ccRCC. Considering that the current study is retrospective, these findings' impact on clinical practice needs to be further verified in future validation studies.

## Introduction

Renal cell cancer (RCC) is one of the worldwide common carcinomas, with approximately 403,262 new cases and 175,098 deaths expected in 2018^1^. At the time of diagnosis, about 20-30% patients are presented with metastatic disease, namely metastatic RCC (mRCC)^2^. During an era when mRCC was treated with cytokines, a 31% decrease in the risk of mortality and significantly improved median overall survival (OS; 13.6 vs 7.8 months) have attributed to cytoreductive nephrectomy among patients with mRCC^3^. Although succedent targeted therapies have resulted in the progression free survival (PFS) and/or OS benefit, 75-100% of mRCC patients still have had prior nephrectomy^4^. However, it is unknown whether the beneficial effect in the target therapy era could further be improved from cytoreductive nephrectomy (CN) combined with lymph node dissection (LND) in the setting of mRCC. There is growing need for evidence on the indications, safety and outcomes of LND in patients with RCC. The role of LND in RCC patients is controversial, and related literature were limited. A systematic review discussing the LND in RCC concluded that the extent of the most commonly dissected templates might be insufficient to catch the overall anatomic pattern of lymphatic drainage from RCC due to the unpredictable renal lymphatic anatomy and the evidence from available prospective mapping studies^5^. Similarly, another systematic review reported that indication and techniques of LND are still controversial for upper tract urothelial carcinoma (UTUC)^6^.

Based on this consideration, we evaluated whether LND at cytoreductive nephrectomy in mRCC patients might be associated with oncologic outcomes, compared with no LND. To test this hypothesis, we relied on the most contemporary population-based cohort of mRCC patients treated with cytoreductive nephrectomy from within the Surveillance, Epidemiology, and End Result (SEER) database.

## Materials and Methods

### Data source and patient selection

**Figure 1:**
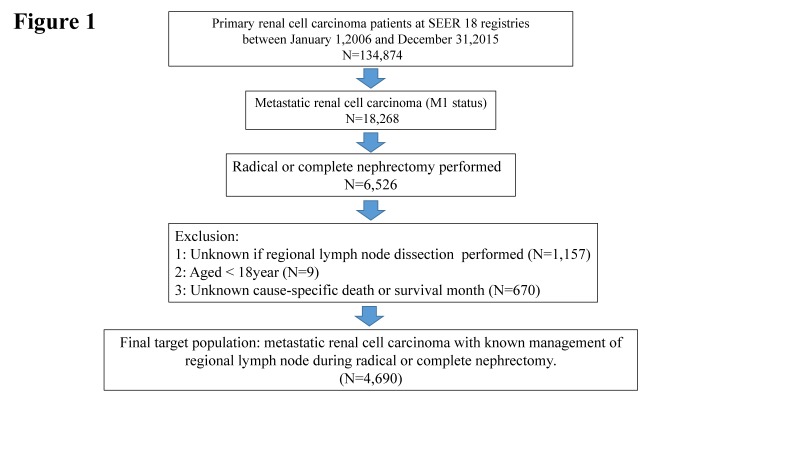
The flowchart of selecting study population. SEER = the Surveillance, Epidemiology, and End Result database.

For the purpose of our study, the study cohort was strictly selected from the Surveillance, epidemiology, and End Results (SEER) database, which encompasses approximately 28% of the American population. As shown in Fig 1, the SEER-stat software (SEER* 8.3.4) was used to extract target population: primary renal cancer patients (international Classification of Disease for Oncology [ICD-O-3], site code C64.9) diagnosed between January 1, 2006 and December 31, 2015 (N = 134,874) based on target therapy started being commonly used for kidney cancer in US after 2006. Moreover, metastatic renal cancer patients underwent radical or complete nephrectomy (N = 6,526) were qualified for further analyses. Included histological subtypes were: clear cell RCC, papillary, chromophobe, sarcomatoid, cyst-associated RCC, collecting duct carcinoma, and any RCC. Cancer specific survival (CSS) was defined according to the SEER mortality code. Further exclusion criteria were lack of data regarding LND (n = 1157), and age < 18 years (n = 9) and unknown cancer-specific death or survival month (n=670). All patients had available data on follow-up. These selection criteria yielded 4690 patients **(Fig.1)**.

### Covariates

As shown in Table 1, age at diagnosis was divided into 18-55 years, 56-65 years and 66-93 years. T-stage was defined as T1-T2, T3-T4 and TX. Likely, N-stage was record as N0, N1 and NX while Grade was divided into I-II, III-IV and “Unknown”. Tumor size were categorized based on the interquartile rang (<7.0cm, 7.0-9.0cm, 9.1-11.9cm, ≥12.0cm and unknown). Year of diagnosis was separated into 2006-2010 and 2011-2015 for further analyses. Race was classified as white, black, other and unknown. And tumor location was described as “Left kidney”, “Right kidney” and “Other” while marital status consisted of “Married”, “Single/Unmarried”, “Divorced/Separated”, “Widowed” and “Unknown”. Based on International Classification of Diseases for oncology, 3rd Edition (ICD-O-3), clear cell RCC (ccRCC) (8310, 8320 and 8316) was distinguished from non-ccRCC. And procedure of surgery of reginal LND was described as “Performed” and “Not performed”.

### Statistical analysis

Medians and interquartile ranges (IQR), as well as frequencies and proportions were reported for continuous and categorical variables, respectively. The difference between groups was compared using x2 test or Fisher's exact test for categorical variables and t test for numerical variables. Survival differences between the LND and non-LND groups in each set, and survival differences of patients with different number of positive lymph node (NPN) and with different number of resected lymph node (NRN) were assessed by the Kaplan-Meier estimate and compared using the log rank test. Multivariate Cox regression analysis and data stratification analysis were performed to test the independent prognostic role of LND in predicting CSS. All statistical analyses were performed using the R software (version 2.15.0, www.r-project.org). All statistical tests were two-sided with a level of significance set at *p* value < 0.05.

## Results

Within the SEER database, we identify 4690 patients with newly diagnosed mRCC treated with radical or complete nephrectomy between 2006 and 2015. Of these, 1902 (40.6%) underwent LND and 2788 (59.4%) did not. The clinic-pathologic features were summarized in **Table 1**. Briefly, median follow-up periods were 12 and 16 months for, respectively, LND and non-LND patients. LND patients were younger (p < 0.01), more frequently harbored T3-4 stage (82% vs 66.4%, p < 0.01), N1 stage (50.1% vs 11.9%, p < 0.01) and Grade III-IV (77.2% vs 65.8%, p < 0.01), and had a larger tumor size (p < 0.01), than patients with no LND.

**Figure 2:**
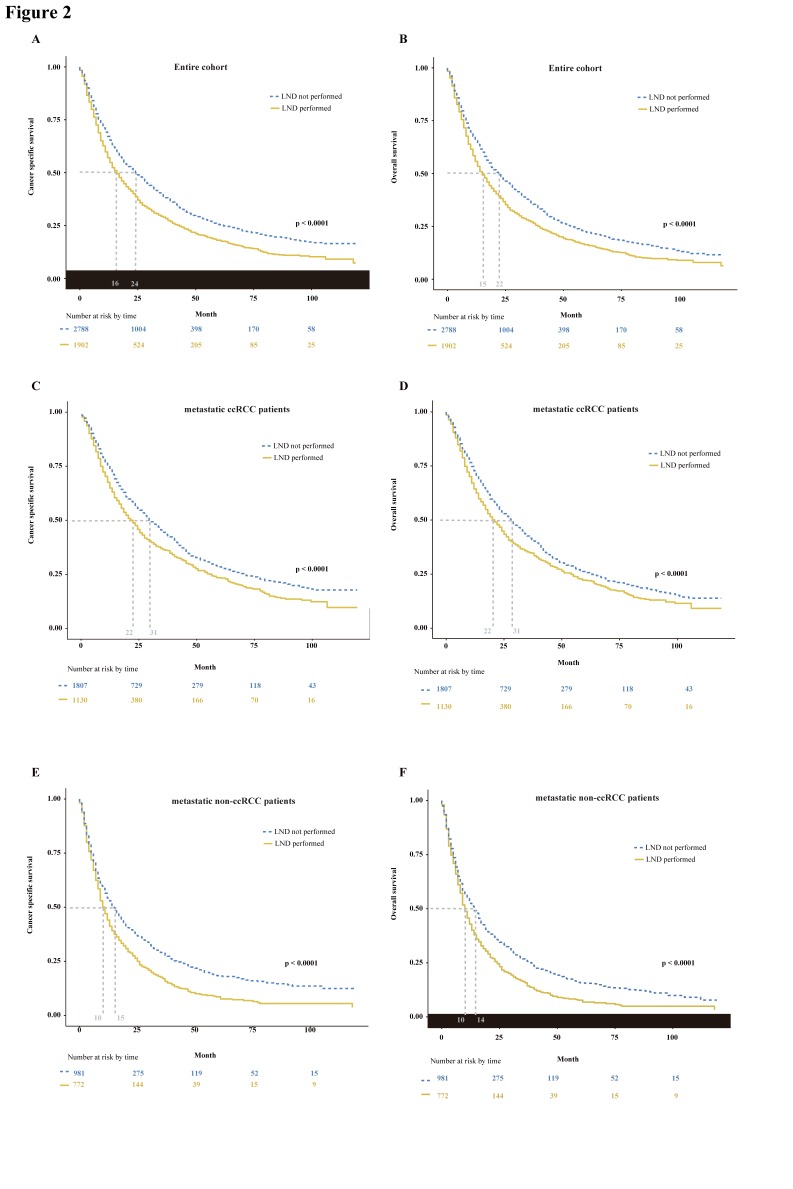
Kaplan-Meier plots depicting CSS (A) and OS (B) in entire cohort, CSS (C) and OS (D) in metastatic ccRCC patients, and CSS (E) and OS (F) in metastatic non-ccRCC patients, stratified according to the presence or absence of LND. CSS = cancer specific survival; OS = overall survival; ccRCC = clear cell renal cell cancer; LND = lymph node dissection.

Trend analysis demonstrated that the number of mRCC patients underwent radical or complete nephrectomy gradually increased from 2006 to 2015 (p < 0.01, **Supplemental Fig. 1A**). And the proportion of LND remained stable during the study period (p = 0.91, **Supplemental Fig. 1B**). In the entire cohort, LND had a destructive effect on CSS and OS. The median CSS of 16 months for mRCC patients who received LND at nephrectomy versus 24 months for mRCC patients who did not (p < 0.0001, **Fig. 2A**). Similarly, the median OS of mRCC patients according to presence or absence of LND was, respectively, 15 versus 22 months (p < 0.0001, **Fig. 2B**). Furthermore, multivariable Cox regression models demonstrated LND at nephrectomy still exhibited an increased risk of damaged effect on CSS (hazard ratio [HR]: 1.18, 95% confidence interval (CI): 1.09-1.27; p < 0.01) and OS (HR: 1.13, 95% CI: 1.05-1.21; p < 0.01) compared with non-LND at nephrectomy in mRCC patients (**Table 2**). Subsequently, we performed additional subgroup analyses according to histological type. In metastatic patients with clear cell RCC (ccRCC), we found the median CSS of 22 versus 31 months (p < 0.0001, **Fig. 2C**) and the median OS of 22 versus 31 months (p < 0.0001, **Fig. 2D**) according to presence or absence of LND. Meanwhile, the damaged effects of LND on CSS and OS in metastatic non-ccRCC patients were also detected (CSS: 10 versus 15 months, p < 0.0001, **Fig. 2E**; OS: 10 versus 14 months, p < 0.0001, **Fig. 2F**).

**Figure 3:**
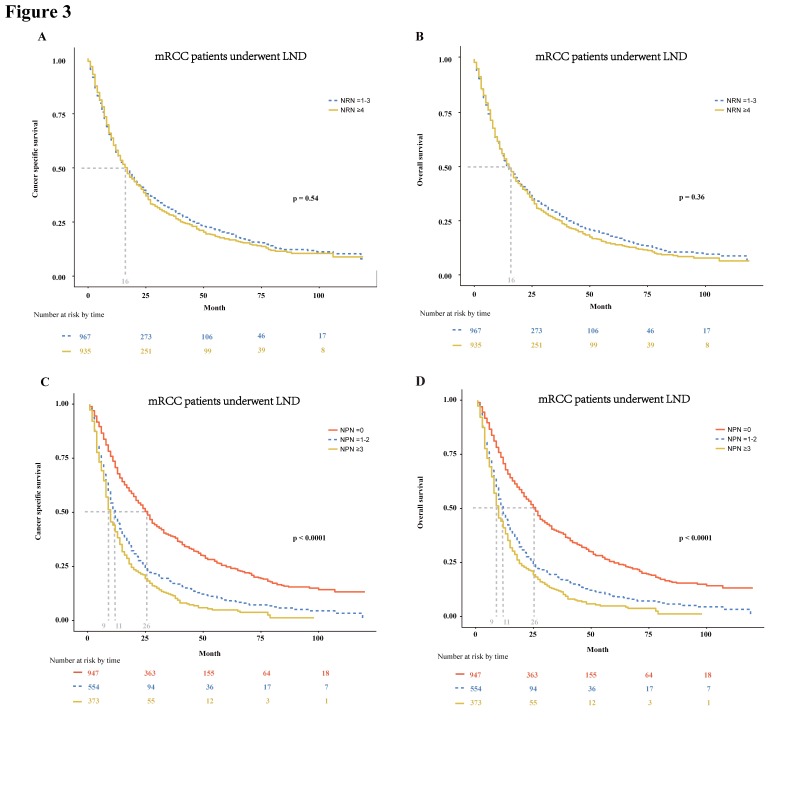
In mRCC patients treated with LND at cytoreductive nephrectomy, Kaplan-Meier plots depicting CSS (A) and OS (B) stratified by NRN, and CSS (C) and OS (D) stratified by NPN. mRCC = metastatic renal cell cancer; LND = lymph node dissection; CSS = cancer specific survival; OS = overall survival; NRN = the number of resected lymph node; NPN = the number of positive lymph node.

In patients with mRCC treated with LND, we further tested the effect of the NRN and NPN on CSS and OS. We found that the NRN had no effect on both CSS and OS (p = 0.54 and 0.36, respectively; **Fig. 3A-B**). In multivariable Cox regression models, the NRN still exhibited no effect on CSS (HR: 0.98, 95% CI: 0.88-1.10; p = 0.68; **Table 3**) and OS (HR: 1.00, 95% CI: 0.89-1.11; p = 0.93; **Table 3**). However, the NPN was found to exert effect on CSS and OS, with results as follows: the median CSS of 26 months for patients with NPN equal to zero versus 11months for patients with NPN ≤ 2 and 9 months for patients with NPN ≥ 3 (p < 0.0001, **Fig. 3C**), the median OS of 26 months for patients with NPN equal to zero versus 11months for patients with NPN ≤ 2 and 9 months for patients with NPN ≥ 3 (p < 0.0001,** Fig.3D**). The effect of NPN on CSS and OS were also confirmed in multivariable Cox regression models (CSS: HR = 1.60-1.85, p < 0.01; OS: HR = 1.58-1.85, p < 0.01; **Table 3**).

## Discussion

In the current study, we found that LND at nephrectomy had a damaged effect on oncologic outcomes both in the overall cohort and in subgroups of metastatic patients with ccRCC and non-ccRCC. And we also identified that oncologic benefit could not be derived from the extent of LND among mRCC patients underwent LND at nephrectomy. Taken together, these findings suggested that LND at nephrectomy conferred a therapeutic harm for M1patients.

In several urologic tumors, LND seemed to improve survival. There was growing evidence that an extensive LND may offer a survival advantage in patients with lymph-node negative and positive bladder cancer^7^. Meanwhile, the survival benefits that LND at radical prostatectomy was related with lower cancer specific mortality and overall mortality were obtained in the setting of metastatic prostate cancer^8^. Based on the premise that complete resection or cytoreductive surgery may improve response to systemic therapy and overall oncologic outcomes, the rationale for a potential oncological benefit to LND was generated^9^. Although no survival advantage of a complete LND in conjunction with a radical nephrectomy was found in N0M0 patients with RCC^10^. Observational data before the era of target therapy have suggested a survival advantage to LND in higher-risk patients (ie, enlarged lymph nodes)^11^-^14^.

Recently, Feuerstein and colleagues^15^ concluded that LND carried out during cytoreductive nephrectomy is not associated with a survival benefit. Whether the extent of LND impacts oncologic outcomes was evaluated in several studies. One population-based study^16^ reported improved CSS with increased lymph node yield among node-positive patients. Another study^17^ demonstrated improved CSS with a greater extent of LND among patients with pT2 tumors, pT3c-pT4 tumors, or tumors with sarcomatoid features.

Interesting, we identified a damaged effect of LND at cytoreductive nephrectomy on CSS and OS in the SEER database (2006-2015) selected according to target therapy started being commonly used in the US after 2006. And the NRN was not associated with oncological outcomes. These results were not consistent with previous studies^15^-^17^. Although the finding that the NPN was related with worse prognosis in our study was supported by other researches^15^, ^18^, LND for providing important staging information should be cautiously performed in mRCC patients. For RCC patients with clinically negative lymph nodes, the indication for LND together with partial nephrectomy or RN is still controversial^14^. And according to the EAU guideline, in patients with localised disease without evidence of lymph node metastases, a survival advantage of LND in conjunction with RN is not demonstrated in randomized trails^19^. When it comes to the treatment of locally advanced RCC patients with clinically positive LNs (cN+), LND is always justified^20^. However, the extent of LND remains controversial^11^. And the EAU guidelines suggest that in patients with locally advancer disease due to clinically enlarged lymph nodes, the survival benefit of LND is unclear but LND can add staging information^19^. Nevertheless, the EAU guideline failed to provide suggestions in the aspect that whether LND should be performed for mRCC patients. There are several potential explanations to reconcile the different findings. Firstly, LND at nephrectomy in mRCC may translate into higher early complication rates, and the CSS and OS benefits associated with LND at nephrectomy may be undermined by higher rates of adverse early postoperative outcomes. It is also possible that earlier administration of target therapy for mRCC patients may have mitigated the benefit of LND at the time of nephrectomy^21^. More importantly, mapping studies^22^ detected direct lympho-venous communications to the renal vein and inferior vena cava, and therefore RCC may be less likely to have a prolonged loco-regional phase and enhanced local control in the retroperitoneum may not translate into a survival benefit. In conclusion, the benefits and harms of LND for mRCC patients are difficult to report given the low quality of most available series, especially the lack of granular data on surgical quality metrics.

Despite the novelty of our findings, limitations need to be acknowledged. Firstly, our study was based on the data from SEER database which did not provide sufficient individuals' information, such as patients' performance status, number and specific locations of metastases, type of systemic therapies (ie, target therapy), postoperative complications, as well as other established predictors associated with mRCC patients' survival, so additional sets of independent samples from clinical trials are needed to prospectively confirm our findings. Secondly, because the SEER database do not precisely recorded the approach of LND, the impact of whether template-based LND or not on mRCC patients' outcomes could not evaluated. Thirdly, the choice leading the surgeon to perform or not LND (selection bias) and lack of information on metastatic burden of the disease may influence our findings. Additionally, these results require validation in patients undergoing partial nephrectomy. Moreover, no data regarding type of systemic therapies (ie, target therapy) were available, these variables should also be considered in future analyses. Last but not least, several important information mentioned above, and baseline hematological and/or biochemical blood values that represent established predictors of survival in mRCC patients, were not available in sets of the current study, and further analysis stratified by these features are necessary in the future research.

To the best of our knowledge, we are the first to demonstrate that LND at cytoreductive nephrectomy was associated with lower CSS and OS relative to non-LND in metastatic patients with ccRCC and non-ccRCC. And there was no association of NRN with survival. These findings displayed that LND should not be considered at cytoreductive nephrectomy in the setting of mRCC patients. Considering that the current study is retrospective, these findings' impact on clinical practice needs to be further verified in future validation studies.

## Supplementary Material

Supplementary figures.

## Figures and Tables

**Table 1:** Baseline characteristics of 4690 of metastatic renal cell cancer patients treated with radical nephrectomy within the Surveillance, Epidemiology and End Results database, stratified according to the presence or absence of lymph node dissection.

Characteristic	Overall = 4690 (%)	LND = 1902 (%)	Non-LND = 2788 (%)	*P* value
Follow-up				<0.01
Median (IQR)	14(6-32)	12(5-26)	16(6-35)	
Age at diagnosis				<0.01
18-55	1572(33.5)	743(39.1)	829(29.7)	
56-65	1590(33.9)	635(33.4)	955(34.3)	
66-93	1528(32.6)	524(27.5)	1004(36)	
T-stage				<0.01
T1-T2	1240(26.4)	336(17.7)	904(32.4)	
T3-T4	3410(72.7)	1559(82.0)	1851(66.4)	
TX	40(0.9)	7(0.4)	33(1.2)	<0.01
N-stage				
N0	3189(68.0)	944(49.6)	2245(80.5)	
N1	1285(27.4)	952(50.1)	333(11.9)	
NX	216(4.6)	6(0.3)	210(7.5)	
Grade				<0.01
I-II	839(17.9)	229(12.0)	610(21.9)	
III-IV	3302(70.4)	1468(77.2)	1834(65.8)	
Unknown	549(11.7)	205(10.8)	344(12.3)	
Tumor size				<0.01
<7.0cm	1185(25.3)	366(19.2)	819(29.4)	
7.0-9.0cm	1219(26)	432(22.7)	787(28.2)	
9.1-11.9cm	1082(23.1)	474(24.9)	608(21.8)	
≥12.0cm	1149(24.5)	618(32.5%)	531(19.0)	
unknown	55(1.2)	12(0.6)	43(1.5)	
Year of diagnosis				0.71
2006-2010	2253(48.0)	920(48.4)	1333(47.8)	
2011-2015	2437(52.0)	982(51.6)	1455(52.2)	
Gender				0.14
Male	3303(70.4)	1317(69.2)	1986(71.2)	
Female	1387(29.6)	585(30.8)	802(28.8)	
Race				0.56
White	3960(84.4)	1598(84.0)	2362(84.7)	
Black	357(7.6)	157(8.3)	200(7.2)	
Other Unknown	363(7.7)	143(7.5)	220(7.9)	
	10(0.2)	4(0.2)	6(0.2)	
Tumor location				<0.01
Left kidney	2428(51.8)	1094(57.5)	1334(47.8)	
Right Kidney	2246(47.9)	803(42.2)	1443(51.8)	
Other	16(0.3)	5(0.3)	11(0.4)	
Marital status				<0.01
Married	3075(65.6)	1257(66.1)	1819(65.2)	
Single/Unmarried	665(14.2	281(14.8)	303(10.8)	
Divorced/Separated	490(10.4)	188(9.9%)	384(13.8)	
Widowed	295(6.3)	114(6.0)	181(6.5)	
Unknown	164(3.5)	62(3.3)	102(3.7)	

LND = lymph node dissection; IQR = interquartile ranges.

**Table 2:** Multivariable Cox regression models predicting cancer specific survival and overall survival in patients with metastatic renal cell cancer (RCC) underwent radical or complete nephrectomy within the Surveillance, Epidemiology and End Results database.

Variables	Cancer specific survival		Overall survival	
	HR(95% CI)	*P* value	HR(95% CI)	*P* value
LND				
Not-Performed	Ref.		Ref.	
Performed	1.18(1.09-1.27)	<0.01	1.13(1.05-1.21)	<0.01
Histology				
clear cell RCC	Ref.		Ref.	
non-clear cell RCC	1.64(1.52-1.76)	<0.01	1.55(1.44-1.67)	<0.01
T-stage				
T1-T2	Ref.		Ref.	
T3-T4	1.37(1.25-1.50)	<0.01	1.23(1.22-1.45)	<0.01
TX	1.19(0.73-1.95)	0.48	1.29(0.83-2.02)	<0.01
Grade				
I-II	Ref.		Ref.	
III-IV	1.68(1.51-1.87)	<0.01	1.77(1.59-1.92)	<0.01
Unknown	1.37(1.18-1.58)	<0.01	1.48(1.07-2.04	<0.01
Size (cm)				
0-69	Ref.		Ref.	
70-90	1.18(1.06-1.30)	<0.01	1.13(1.02-1.25)	0.02
91-119	1.11(1.01-1.24)	0.05	1.04(0.94-1.16)	0.43
>120	1.29(1.17-1.44)	<0.01	1.21(1.09-1.34)	<0.01
Age				
18-55	Ref.		Ref.	
56-65	1.03(0.95-1.13)	0.46	1.04(0.95-1.13)	0.40
66-93	1.17(1.07-1.29)	<0.01	1.22(1.12-1.34)	<0.01
Gender				
Male	Ref.		Ref.	
Female	1.09(1.00-1.176)	0.05	1.05(0.97-1.14)	0.20
Year of diagnosis				
2006-2010	Ref.		Ref.	
2011-2015	0.95(0.88-1.02)	0.14	0.93(0.86-1.00)	0.05
Race				
White	Ref.		Ref.	
Black	1.18(1.03-1.36)	<0.01	1.25(1.10-1.42)	<0.01
Other	0.91(0.80-1.04)	0.18	0.92(0.80-1.05)	0.20
Marital status				
Married	Ref.		Ref.	
Divorced/Separated	1.05(0.93-1.18)	0.44	1.06(0.94-1.18)	0.35
Single/unmarried	0.99(0.85-1.16)	0.92	1.12(1.01-1.25)	0.03
Widowed	0.95(0.77-1.17)	0.65	1.05(0.90-1.22)	0.51

LND = lymph node dissection; HR = hazard ratio; CI = confidence interval; Ref. = reference.

**Table 3:** Multivariable Cox regression models predicting cancer specific survival and overall survival in patients with metastatic renal cell cancer underwent lymph node dissection (LND) at radical nephrectomy within the Surveillance, Epidemiology and End Results database ^a^.

Variables	Cancer specific survival		Overall survival	
	HR(95% CI)	*p* value	HR(95% CI)	*p* value
NRN after LND				
1-3	Ref.		Ref.	
≥4	0.98(0.88-1.10)	0.68	1.00(0.89-1.11)	0.93
NPN after LND				
0	Ref.		Ref.	
1	1.69(1.46-1.96)	<0.01	1.67(1.45-1.92)	<0.01
2	1.60(1.32-1.94)	<0.01	1.58(1.31-1.91)	<0.01
≥3	1.85(1.57-2.17)	<0.01	1.85(1.58-2.16)	<0.01

NRN = number of resected lymph node; NPN = number of positive lymph node; HR = hazard ratio; CI = confidence interval; Ref. = reference. ^a^ Adjusted for age, sex, race, marital status, year of diagnosis, tumor laterality, histology, T-stage, Grade and tumor size.

## References

[1] Bray F, Ferlay J, Soerjomataram I (2018). Global cancer statistics 2018: globocan estimates of incidence and mortality worldwide for 36 cancers in 185 countries[J]. CA Cancer J Clin.

[2] Gupta K, Miller JD, Li JZ (2008). Epidemiologic and socioeconomic burden of metastatic renal cell carcinoma (mrcc): a literature review[J]. CANCER TREAT REV.

[3] Flanigan RC, Mickisch G, Sylvester R (2004). Cytoreductive nephrectomy in patients with metastatic renal cancer: a combined analysis[J]. J Urol.

[4] Bhindi B, Abel EJ, Albiges L (2019). Systematic review of the role of cytoreductive nephrectomy in the targeted therapy era and beyond: an individualized approach to metastatic renal cell carcinoma[J]. EUR UROL.

[5] Campi R, Sessa F, Di Maida F (2018). Templates of lymph node dissection for renal cell carcinoma: a systematic review of the literature[J]. Front Surg.

[6] Campi R, Minervini A, Mari A (2017). Anatomical templates of lymph node dissection for upper tract urothelial carcinoma: a systematic review of the literature[J]. Expert Rev Anticancer Ther.

[7] Stein JP, Skinner DG (2005). The role of lymphadenectomy in high-grade invasive bladder cancer[J]. Urol Clin North Am.

[8] Mazzone E, Preisser F, Nazzani S (2018). The effect of lymph node dissection in metastatic prostate cancer patients treated with radical prostatectomy: a contemporary analysis of survival and early postoperative outcomes[J].

[9] Margulis V, Wood CG (2008). The role of lymph node dissection in renal cell carcinoma: the pendulum swings back[J]. CANCER J.

[10] Blom JH, van Poppel H, Marechal JM (2009). Radical nephrectomy with and without lymph-node dissection: final results of european organization for research and treatment of cancer (eortc) randomized phase 3 trial 30881[J]. EUR UROL.

[11] Capitanio U, Becker F, Blute ML (2011). Lymph node dissection in renal cell carcinoma[J]. EUR UROL.

[12] Herrlinger A, Schrott KM, Schott G (1991). What are the benefits of extended dissection of the regional renal lymph nodes in the therapy of renal cell carcinoma[J]. J Urol.

[13] Schafhauser W, Ebert A, Brod J (1999). Lymph node involvement in renal cell carcinoma and survival chance by systematic lymphadenectomy[J]. ANTICANCER RES.

[14] Bekema HJ, MacLennan S, Imamura M (2013). Systematic review of adrenalectomy and lymph node dissection in locally advanced renal cell carcinoma[J]. EUR UROL.

[15] Feuerstein MA, Kent M, Bernstein M (2014). Lymph node dissection during cytoreductive nephrectomy: a retrospective analysis[J]. INT J UROL.

[16] Whitson JM, Harris CR, Reese AC (2011). Lymphadenectomy improves survival of patients with renal cell carcinoma and nodal metastases[J]. J Urol.

[17] Capitanio U, Suardi N, Matloob R (2014). Extent of lymph node dissection at nephrectomy affects cancer-specific survival and metastatic progression in specific sub-categories of patients with renal cell carcinoma (rcc)[J]. BJU INT.

[18] Feuerstein MA, Kent M, Bazzi WM (2014). Analysis of lymph node dissection in patients with &gt;/=7-cm renal tumors[J]. WORLD J UROL.

[19] Eau guidelines (2018). Edn.

[20] Tsui KH, Shvarts O, Smith RB (2000). Prognostic indicators for renal cell carcinoma: a multivariate analysis of 643 patients using the revised 1997 tnm staging criteria[J]. J Urol.

[21] Boorjian SA, Crispen PL, Lohse CM (2008). Surgical resection of isolated retroperitoneal lymph node recurrence of renal cell carcinoma following nephrectomy[J]. J UROLOGY.

[22] Karmali RJ, Suami H, Wood CG (2014). Lymphatic drainage in renal cell carcinoma: back to the basics[J]. BJU INT.

