# Supplementary figures and images for: Adverse Effect of Lymph Node Dissection in Metastatic Renal Cell Cancer Patients Treated with Cytoreductive Nephrectomy: A Contemporary Analysis of Survival
Source: J Cancer. 2019 Aug 8;10(19):4639–46. doi: 10.7150/jca.33923 (PMC6746138; doi:10.7150/jca.33923)

# Supplemental Figure S1

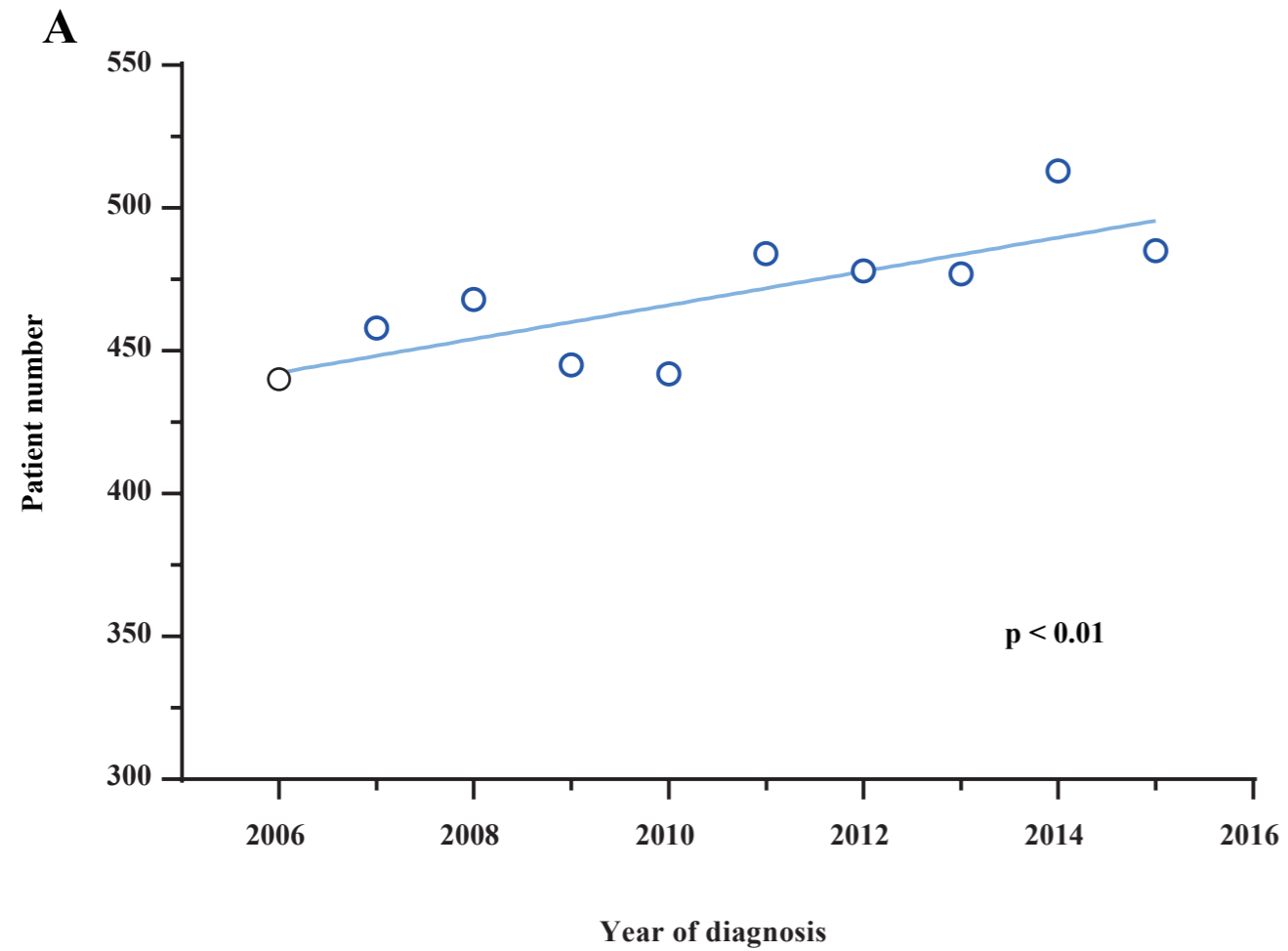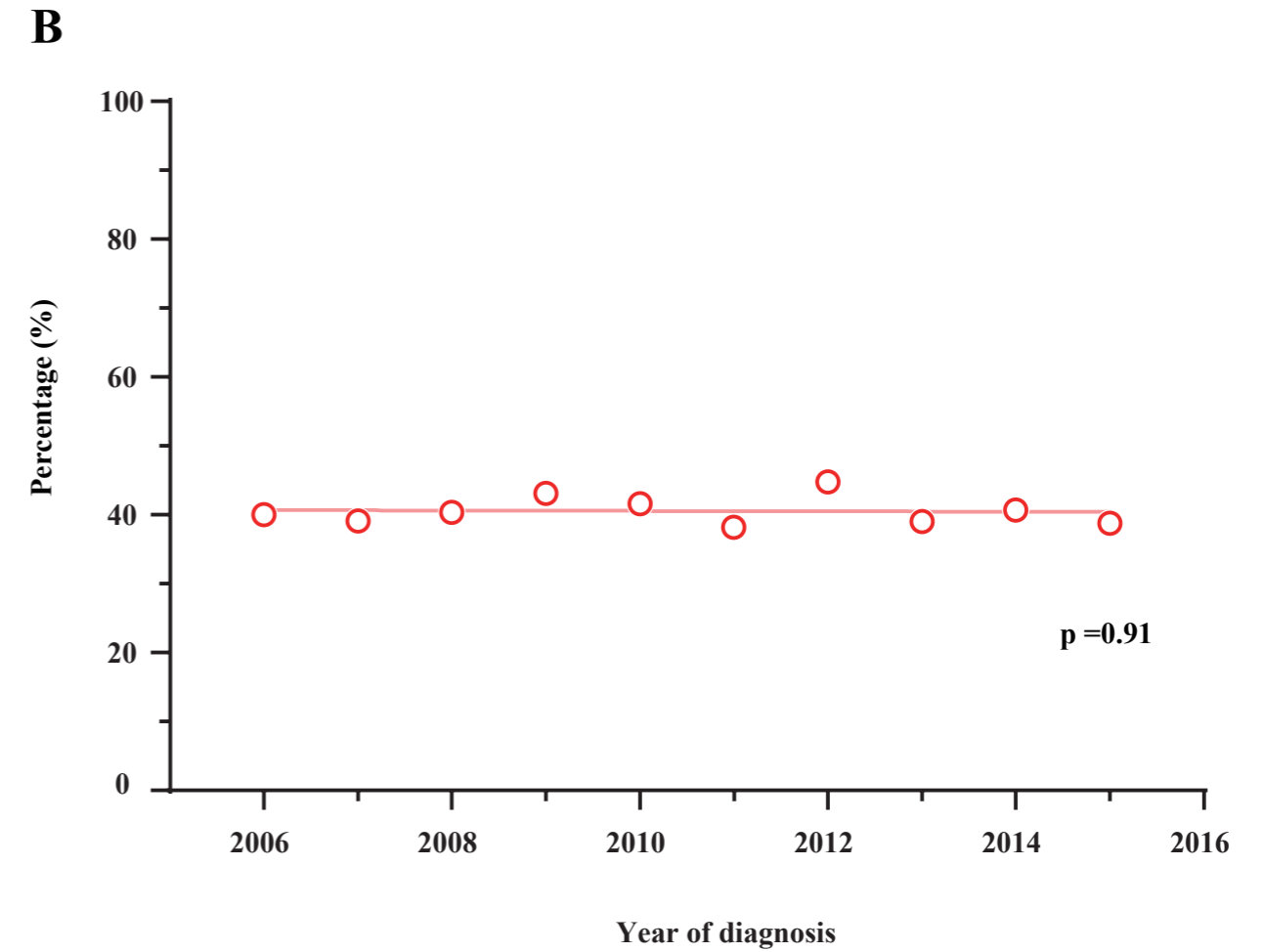

Supplement: Supplementary file 1 — Supplementary figures. [file jcav10p4639s1.pdf]
